# (3R*)-Methyl 3-[(2S*)-4,6-dimethoxy-2-(4-methoxyphenyl)-3-oxo-2,3-dihydro-1-benzofuran-2-yl]-2-methoxycarbonyl-3-phenylpropionate

**DOI:** 10.1107/S1600536809042196

**Published:** 2009-10-28

**Authors:** Xian-You Wang, Nan Li, Yong-Qiang Ma, Zhao-Hai Qin

**Affiliations:** a College of Science, China Agricultural University, Beijing 100094, People’s Republic of China

## Abstract

The title compound, C_29_H_28_O_9_, was isolated from the reaction of 4,6-dimeth­oxy-2-(4-methoxy­phen­yl)-3-benzofuran and α-methoxy­carbonyl­cinnaminate. The two aromatic rings form a dihedral angle of 22.7 (1)°. One methoxy­carbonyl group is disordered between two orientations in a 0.612 (4):0.388 (4) ratio. The crystal structure exhibits no significantly short inter­molecular contacts.

## Related literature

The title compound is a key inter­mediate in the synthesis of rocaglamide, see: Kraus & Sy (1989 ▶); Li *et al.* (2008 ▶). For the biological activity of rocaglamide derivatives, see: Zhu *et al.* (2007 ▶).
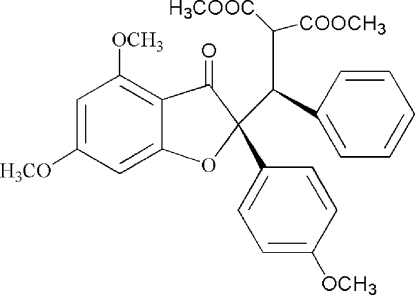


## Experimental

### 

#### Crystal data

C_29_H_28_O_9_
*M* *_r_* = 520.51
Triclinic, 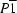

*a* = 6.901 (5) Å
*b* = 10.478 (5) Å
*c* = 18.467 (5) Å
α = 79.838 (5)°
β = 86.976 (5)°
γ = 77.097 (5)°
*V* = 1281.1 (12) Å^3^
*Z* = 2
Cu *K*α radiation
μ = 0.84 mm^−1^
*T* = 295 K
0.40 × 0.36 × 0.34 mm

#### Data collection

Oxford Diffraction Gemini S Ultra diffractometer
Absorption correction: multi-scan (*CrysAlis Pro*; Oxford Diffraction, 2009 ▶). *T*_min_ = 0.731, *T*_max_ = 0.764
24595 measured reflections
4811 independent reflections
4581 reflections with *I* > 2σ(*I*)
*R*_int_ = 0.018

#### Refinement

*R*[*F* ^2^ > 2σ(*F* ^2^)] = 0.042
*wR*(*F* ^2^) = 0.093
*S* = 1.01
4811 reflections
378 parameters
9 restraints
H-atom parameters constrained
Δρ_max_ = 0.41 e Å^−3^
Δρ_min_ = −0.45 e Å^−3^

### 

Data collection: *CrysAlis CCD* (Oxford Diffraction, 2009 ▶); cell refinement: *CrysAlis RED* (Oxford Diffraction, 2009 ▶); data reduction: *CrysAlis RED*; program(s) used to solve structure: *SHELXS97* (Sheldrick, 2008 ▶); program(s) used to refine structure: *SHELXL97* (Sheldrick, 2008 ▶); molecular graphics: *ORTEP-3* (Farrugia, 1997 ▶); software used to prepare material for publication: *SHELXL97*.

## Supplementary Material

Crystal structure: contains datablocks I, global. DOI: 10.1107/S1600536809042196/cv2618sup1.cif

Structure factors: contains datablocks I. DOI: 10.1107/S1600536809042196/cv2618Isup2.hkl

Additional supplementary materials:  crystallographic information; 3D view; checkCIF report

## Figures and Tables

**Fig. 1.**
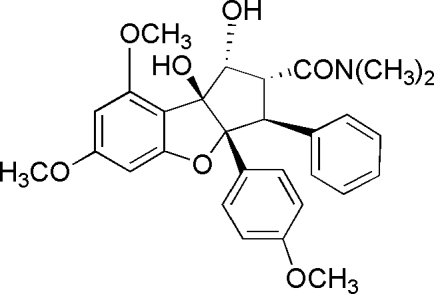
Rocaglamide.

**Fig. 2.**
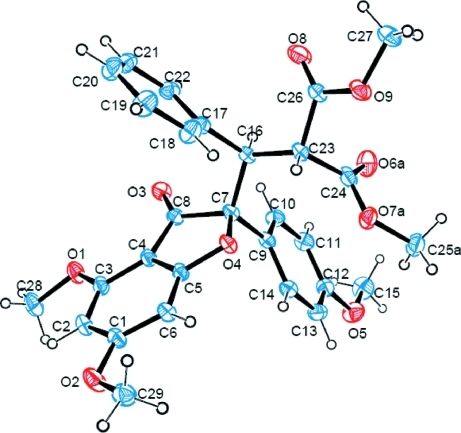
The molecular structure of (I), showing the labelling scheme. Displacement ellipsoids are drawn at the 50% probability level for all non-H atoms. Only major parts of the disordered atoms are shown.

## supplementary crystallographic information

### Comment

Rocaglamide (Fig. 1), featuring a cyclopenta[*b*]benzofuran ring system, was first isolated from the roots and stems of *Aglia elliptifolia* by King  and co-workers in 1982. Since then, rocaglamide and related compounds have shown cytostatic and cytotoxic activity against a variety of human cancer cell lines (King *et al.*, 1982; Zhu *et al.*, 2007). The structural complexity of rocaglamide and its significant activity make it an attractive synthetic target. To date, several synthetic routes were developed for rocaglamide and its derivatives (Kraus *et al.*, 1989; Li *et al.*, 2008).

The title compound (I) is one of the key intermediates to rocaglamide in Li's strategy (Li *et al.*, 2008). Preparation of the compound involved the reaction of 4,6-dimethoxy-2-(4-methoxyphenyl)-3-benzofuran, α-methoxycarbonylcinnaminate and Triton B (*N*,*N*,*N*-trimethylbenzylammonium hydroxide) for 4 h with concomitant stirring in 40 °C and purification by silica-gel column chromatography (petroleumether/EtOAc, 3:1) to give colourless crystals.

In (I) (Fig. 2), two aromatic rings form a dihedral angle of 22.7 (1) °, and one methoxycarbonyl group is disordered between two orientations in an approximate ratio 3:2. The X-ray crystal structure confirms that the substance produced is a racemic mixture of Methyl 2-methoxycarbonyl-(3*R*, 4S) - and - (3*S*, 4*R*) - 4-(4, 6-dimethoxy-3-oxo -2, 3-dihydrobenzofuran-2-yl]-4-(4-methoxy-phenyl)-3-phenylpropionate as predicted by NOESY NMR experiments.

### Experimental

Under N_2_ atmosphere, to asolution of 4, 6-dimethoxy- 2-(4-methoxyphenyl)-3- benzofuran (3.0 g, 10 mmol) in anhydrous THF (100 ml) was added a solution of Triton B (40% in CH_3_OH, 0.3 ml) and a solution of α-methoxycarbonylcinnaminate(3.2 g, 14.5 mmol) in anhydrous THF (40 ml) by syringe. After stirring at 40 °C for 4 h, the solvent was removed *in vacuo*. To the residue was added a solution of HCl (1 mol/*L*, 35 ml), and this solution was extracted with CH_2_Cl_2_ (3 × 40 ml). The combined organic phase was washed with saturated NaCl solution (2 × 30 ml), dried with Na_2_SO_4_, and concentrated. The crude product was separated by silica-gel columnchromatography (petroleum ether/EtOAc, 3:1) to afford colourless crystal of compound (I).

### Refinement

All H atoms were positioned geometrically with C—H = 0.93 - 0.98 Å and constrained to ride on their parent atoms, with *U*_iso_(H) = *xU*_eq_(C), where *x* = 1.5 for methyl H and *x *= 1.2 for other H atoms.

### Crystal data

**Table:**

C_29_H_28_O_9_	*Z* = 2
*M**_r_* = 520.51	*F*(000) = 548
Triclinic, *P*1	*D*_x_ = 1.349 Mg m^−^^3^
*a* = 6.901 (5) Å	Cu *K*α radiation, λ = 1.54184 Å
*b* = 10.478 (5) Å	Cell parameters from 21851 reflections
*c* = 18.467 (5) Å	θ = 2.4–72.1°
α = 79.838 (5)°	µ = 0.84 mm^−^^1^
β = 86.976 (5)°	*T* = 295 K
γ = 77.097 (5)°	Block, colourless
*V* = 1281.1 (12) Å^3^	0.40 × 0.36 × 0.34 mm

### Data collection

**Table:**

Oxford Diffraction Gemini S Ultra diffractometer	4811 independent reflections
Radiation source: Enhance Ultra (Cu) X-ray Source	4581 reflections with *I* > 2σ(*I*)
mirror	*R*_int_ = 0.018
Detector resolution: 15.9149 pixels mm^-1^	θ_max_ = 70.1°, θ_min_ = 2.4°
ω scans	*h* = −8→6
Absorption correction: multi-scan (*CrysAlis PRO*; Oxford Diffraction, 2009).	*k* = −12→12
*T*_min_ = 0.731, *T*_max_ = 0.764	*l* = −22→22
24595 measured reflections	

### Refinement

**Table:**

Refinement on *F*^2^	Secondary atom site location: difference Fourier map
Least-squares matrix: full	Hydrogen site location: inferred from neighbouring sites
*R*[*F*^2^ > 2σ(*F*^2^)] = 0.042	H-atom parameters constrained
*wR*(*F*^2^) = 0.093	*w* = 1/[σ^2^(*F*_o_^2^) + (0.0289*P*)^2^ + 0.6245*P*] where *P* = (*F*_o_^2^ + 2*F*_c_^2^)/3
*S* = 1.00	(Δ/σ)_max_ = 0.001
4811 reflections	Δρ_max_ = 0.41 e Å^−^^3^
378 parameters	Δρ_min_ = −0.45 e Å^−^^3^
9 restraints	Extinction correction: *SHELXL*, Fc^*^=kFc[1+0.001xFc^2^λ^3^/sin(2θ)]^-1/4^
Primary atom site location: structure-invariant direct methods	Extinction coefficient: 0.0365 (14)

### Special details

**Table:**

Geometry. All e.s.d.'s (except the e.s.d. in the dihedral angle between two l.s. planes) are estimated using the full covariance matrix. The cell e.s.d.'s are taken into account individually in the estimation of e.s.d.'s in distances, angles and torsion angles; correlations between e.s.d.'s in cell parameters are only used when they are defined by crystal symmetry. An approximate (isotropic) treatment of cell e.s.d.'s is used for estimating e.s.d.'s involving l.s. planes.
Refinement. Refinement of *F*^2^ against ALL reflections. The weighted *R*-factor *wR* and goodness of fit *S* are based on *F*^2^, conventional *R*-factors *R* are based on *F*, with *F* set to zero for negative *F*^2^. The threshold expression of *F*^2^ > σ(*F*^2^) is used only for calculating *R*-factors(gt) *etc*. and is not relevant to the choice of reflections for refinement. *R*-factors based on *F*^2^ are statistically about twice as large as those based on *F*, and *R*- factors based on ALL data will be even larger.

### Fractional atomic coordinates and isotropic or equivalent isotropic displacement parameters (Å^2^)

**Table:**

	*x*	*y*	*z*	*U*_iso_*/*U*_eq_	Occ. (<1)
O1	0.72974 (16)	−0.00047 (10)	0.17466 (6)	0.0477 (3)	
O2	0.11720 (18)	0.08988 (12)	0.05005 (7)	0.0560 (3)	
O3	0.74032 (15)	0.21665 (10)	0.26672 (6)	0.0441 (3)	
O4	0.23465 (13)	0.35531 (9)	0.22326 (5)	0.0339 (2)	
O5	0.1979 (2)	0.09372 (13)	0.55766 (6)	0.0616 (3)	
O8	0.47464 (18)	0.74181 (12)	0.28233 (9)	0.0681 (4)	
O9	0.14431 (16)	0.81607 (10)	0.27886 (7)	0.0517 (3)	
C1	0.2267 (2)	0.12553 (15)	0.09905 (8)	0.0406 (3)	
C2	0.4180 (2)	0.04509 (15)	0.11022 (8)	0.0445 (4)	
H2	0.4594	−0.0263	0.0853	0.053*	
C3	0.5450 (2)	0.07168 (14)	0.15815 (8)	0.0374 (3)	
C4	0.4790 (2)	0.18239 (13)	0.19409 (7)	0.0338 (3)	
C5	0.2860 (2)	0.25550 (12)	0.18279 (7)	0.0320 (3)	
C6	0.1540 (2)	0.23139 (14)	0.13594 (7)	0.0363 (3)	
H6	0.0254	0.2825	0.1295	0.044*	
C7	0.3966 (2)	0.34343 (13)	0.27287 (7)	0.0324 (3)	
C8	0.5705 (2)	0.24013 (13)	0.24547 (7)	0.0334 (3)	
C9	0.3410 (2)	0.27976 (13)	0.34983 (7)	0.0341 (3)	
C10	0.4720 (2)	0.26054 (15)	0.40736 (8)	0.0424 (3)	
H10	0.5905	0.2894	0.3987	0.051*	
C11	0.4297 (2)	0.19920 (16)	0.47741 (8)	0.0469 (4)	
H11	0.5184	0.1883	0.5153	0.056*	
C12	0.2553 (2)	0.15417 (15)	0.49079 (8)	0.0444 (4)	
C13	0.1257 (2)	0.16977 (16)	0.43329 (9)	0.0460 (4)	
H13	0.0095	0.1380	0.4416	0.055*	
C14	0.1678 (2)	0.23218 (14)	0.36369 (8)	0.0392 (3)	
H14	0.0793	0.2424	0.3257	0.047*	
C15	0.3217 (3)	0.0827 (2)	0.61877 (10)	0.0685 (5)	
H15B	0.4527	0.0335	0.6091	0.103*	
H15C	0.2670	0.0374	0.6620	0.103*	
H15A	0.3289	0.1699	0.6263	0.103*	
C16	0.4442 (2)	0.48256 (13)	0.26704 (7)	0.0334 (3)	
H16	0.5424	0.4762	0.3047	0.040*	
C17	0.5380 (2)	0.52433 (13)	0.19253 (8)	0.0347 (3)	
C18	0.4260 (2)	0.57228 (18)	0.12944 (9)	0.0498 (4)	
H18	0.2887	0.5813	0.1324	0.060*	
C19	0.5154 (3)	0.6070 (2)	0.06191 (10)	0.0610 (5)	
H19	0.4378	0.6389	0.0202	0.073*	
C20	0.7178 (3)	0.59466 (18)	0.05638 (10)	0.0566 (4)	
H20	0.7779	0.6175	0.0111	0.068*	
C21	0.8301 (2)	0.54818 (16)	0.11845 (10)	0.0514 (4)	
H21	0.9672	0.5397	0.1151	0.062*	
C22	0.7418 (2)	0.51363 (14)	0.18602 (9)	0.0423 (3)	
H22	0.8203	0.4828	0.2276	0.051*	
C23	0.2594 (2)	0.58820 (13)	0.28276 (8)	0.0362 (3)	
H23	0.1610	0.5978	0.2447	0.043*	
C24	0.1603 (2)	0.56062 (15)	0.35796 (8)	0.0455 (4)	
C26	0.3135 (2)	0.72205 (13)	0.28050 (8)	0.0408 (3)	
C27	0.1644 (3)	0.94722 (15)	0.28982 (12)	0.0638 (5)	
H27B	0.0520	1.0130	0.2692	0.096*	
H27C	0.2841	0.9664	0.2659	0.096*	
H27A	0.1703	0.9484	0.3415	0.096*	
C28	0.7892 (3)	−0.12487 (18)	0.14867 (11)	0.0616 (5)	
H28B	0.7877	−0.1093	0.0959	0.092*	
H28A	0.6986	−0.1806	0.1675	0.092*	
H28C	0.9210	−0.1679	0.1652	0.092*	
C29	−0.0736 (3)	0.17228 (18)	0.03147 (10)	0.0542 (4)	
H29C	−0.1343	0.1365	−0.0037	0.081*	
H29B	−0.0592	0.2603	0.0106	0.081*	
H29A	−0.1558	0.1755	0.0750	0.081*	
O6A	0.2258 (6)	0.5551 (6)	0.41638 (11)	0.0610 (9)	0.612 (4)
O7A	−0.0279 (4)	0.5512 (5)	0.34725 (13)	0.0462 (7)	0.612 (4)
C25A	−0.1494 (4)	0.5308 (3)	0.41401 (13)	0.0527 (8)	0.612 (4)
H25A	−0.2861	0.5443	0.4008	0.079*	0.612 (4)
H25B	−0.1368	0.5929	0.4450	0.079*	0.612 (4)
H25C	−0.1050	0.4418	0.4399	0.079*	0.612 (4)
O6B	0.0009 (7)	0.5422 (10)	0.3754 (4)	0.097 (3)	0.388 (4)
O7B	0.2974 (7)	0.5644 (8)	0.40674 (19)	0.074 (2)	0.388 (4)
C25B	0.2580 (11)	0.5464 (7)	0.4862 (2)	0.090 (2)	0.388 (4)
H25D	0.3803	0.5331	0.5117	0.135*	0.388 (4)
H25E	0.2003	0.4702	0.5007	0.135*	0.388 (4)
H25F	0.1674	0.6239	0.4981	0.135*	0.388 (4)

### Atomic displacement parameters (Å^2^)

**Table:**

	*U*^11^	*U*^22^	*U*^33^	*U*^12^	*U*^13^	*U*^23^
O1	0.0465 (6)	0.0393 (6)	0.0547 (6)	0.0048 (5)	−0.0024 (5)	−0.0187 (5)
O2	0.0601 (7)	0.0565 (7)	0.0579 (7)	−0.0070 (6)	−0.0148 (6)	−0.0299 (6)
O3	0.0363 (6)	0.0397 (6)	0.0555 (6)	−0.0030 (4)	−0.0079 (5)	−0.0104 (5)
O4	0.0358 (5)	0.0298 (5)	0.0365 (5)	−0.0017 (4)	−0.0062 (4)	−0.0120 (4)
O5	0.0730 (8)	0.0697 (8)	0.0418 (6)	−0.0249 (7)	0.0023 (6)	0.0023 (6)
O8	0.0491 (7)	0.0482 (7)	0.1165 (12)	−0.0139 (6)	−0.0003 (7)	−0.0354 (7)
O9	0.0498 (6)	0.0290 (5)	0.0748 (8)	−0.0026 (4)	−0.0009 (5)	−0.0125 (5)
C1	0.0502 (9)	0.0392 (8)	0.0360 (7)	−0.0118 (6)	−0.0038 (6)	−0.0121 (6)
C2	0.0530 (9)	0.0384 (8)	0.0438 (8)	−0.0044 (7)	0.0009 (7)	−0.0193 (6)
C3	0.0410 (8)	0.0316 (7)	0.0379 (7)	−0.0031 (6)	0.0028 (6)	−0.0089 (6)
C4	0.0379 (7)	0.0294 (7)	0.0338 (7)	−0.0055 (5)	0.0006 (5)	−0.0070 (5)
C5	0.0396 (7)	0.0262 (6)	0.0302 (6)	−0.0063 (5)	0.0010 (5)	−0.0065 (5)
C6	0.0398 (7)	0.0338 (7)	0.0352 (7)	−0.0058 (6)	−0.0031 (6)	−0.0081 (6)
C7	0.0344 (7)	0.0290 (6)	0.0343 (7)	−0.0043 (5)	−0.0058 (5)	−0.0083 (5)
C8	0.0366 (7)	0.0270 (6)	0.0352 (7)	−0.0048 (5)	−0.0016 (5)	−0.0039 (5)
C9	0.0398 (7)	0.0267 (6)	0.0359 (7)	−0.0045 (5)	−0.0025 (6)	−0.0083 (5)
C10	0.0442 (8)	0.0438 (8)	0.0408 (8)	−0.0132 (7)	−0.0055 (6)	−0.0057 (6)
C11	0.0538 (9)	0.0487 (9)	0.0382 (8)	−0.0113 (7)	−0.0093 (7)	−0.0047 (7)
C12	0.0558 (9)	0.0377 (8)	0.0383 (8)	−0.0091 (7)	0.0024 (7)	−0.0052 (6)
C13	0.0468 (9)	0.0450 (8)	0.0486 (9)	−0.0160 (7)	0.0019 (7)	−0.0075 (7)
C14	0.0419 (8)	0.0360 (7)	0.0411 (8)	−0.0088 (6)	−0.0047 (6)	−0.0091 (6)
C15	0.0890 (15)	0.0740 (13)	0.0387 (9)	−0.0189 (11)	−0.0037 (9)	0.0023 (8)
C16	0.0363 (7)	0.0289 (6)	0.0356 (7)	−0.0054 (5)	−0.0043 (5)	−0.0083 (5)
C17	0.0377 (7)	0.0272 (6)	0.0400 (7)	−0.0062 (5)	−0.0014 (6)	−0.0083 (5)
C18	0.0398 (8)	0.0637 (10)	0.0437 (8)	−0.0103 (7)	−0.0041 (7)	−0.0032 (7)
C19	0.0608 (11)	0.0768 (13)	0.0413 (9)	−0.0150 (9)	−0.0057 (8)	0.0025 (8)
C20	0.0624 (11)	0.0554 (10)	0.0505 (10)	−0.0165 (8)	0.0127 (8)	−0.0040 (8)
C21	0.0413 (8)	0.0438 (9)	0.0672 (11)	−0.0104 (7)	0.0096 (8)	−0.0061 (8)
C22	0.0389 (8)	0.0344 (7)	0.0522 (9)	−0.0062 (6)	−0.0041 (6)	−0.0046 (6)
C23	0.0401 (8)	0.0305 (7)	0.0387 (7)	−0.0058 (6)	−0.0008 (6)	−0.0103 (6)
C24	0.0602 (10)	0.0313 (7)	0.0444 (9)	−0.0045 (7)	0.0049 (8)	−0.0133 (6)
C26	0.0456 (9)	0.0332 (7)	0.0448 (8)	−0.0066 (6)	−0.0005 (6)	−0.0121 (6)
C27	0.0717 (12)	0.0285 (8)	0.0913 (14)	−0.0093 (8)	0.0114 (10)	−0.0159 (8)
C28	0.0641 (11)	0.0448 (9)	0.0710 (12)	0.0118 (8)	−0.0022 (9)	−0.0268 (9)
C29	0.0510 (10)	0.0614 (11)	0.0568 (10)	−0.0158 (8)	−0.0098 (8)	−0.0207 (8)
O6A	0.074 (2)	0.077 (2)	0.0306 (14)	−0.0115 (17)	0.0055 (14)	−0.0125 (15)
O7A	0.0501 (14)	0.0492 (15)	0.0386 (13)	−0.0111 (9)	0.0131 (10)	−0.0091 (13)
C25A	0.0483 (16)	0.0634 (18)	0.0461 (15)	−0.0120 (13)	0.0125 (12)	−0.0130 (13)
O6B	0.106 (5)	0.065 (3)	0.106 (5)	−0.016 (4)	0.078 (4)	−0.006 (5)
O7B	0.129 (6)	0.050 (2)	0.039 (2)	−0.013 (4)	0.047 (3)	−0.0138 (16)
C25B	0.127 (6)	0.094 (5)	0.057 (3)	−0.035 (4)	0.029 (3)	−0.029 (3)

### Geometric parameters (Å, °)

**Table:**

O1—C3	1.3485 (19)	C16—C23	1.543 (2)
O1—C28	1.4349 (19)	C16—H16	0.9800
O2—C1	1.3589 (18)	C17—C22	1.386 (2)
O2—C29	1.426 (2)	C17—C18	1.387 (2)
O3—C8	1.2141 (18)	C18—C19	1.388 (2)
O4—C5	1.3638 (16)	C18—H18	0.9300
O4—C7	1.4524 (16)	C19—C20	1.374 (3)
O5—C12	1.3665 (19)	C19—H19	0.9300
O5—C15	1.424 (2)	C20—C21	1.371 (3)
O8—C26	1.1788 (18)	C20—H20	0.9300
O9—C26	1.3470 (18)	C21—C22	1.385 (2)
O9—C27	1.4610 (17)	C21—H21	0.9300
C1—C6	1.389 (2)	C22—H22	0.9300
C1—C2	1.402 (2)	C23—C26	1.523 (2)
C2—C3	1.381 (2)	C23—C24	1.525 (2)
C2—H2	0.9300	C23—H23	0.9800
C3—C4	1.4133 (19)	C24—O6B	1.178 (2)
C4—C5	1.386 (2)	C24—O6A	1.1786 (18)
C4—C8	1.4497 (19)	C24—O7B	1.3519 (19)
C5—C6	1.380 (2)	C24—O7A	1.352 (2)
C6—H6	0.9300	C27—H27B	0.9600
C7—C9	1.5280 (19)	C27—H27C	0.9600
C7—C16	1.5491 (19)	C27—H27A	0.9600
C7—C8	1.5545 (19)	C28—H28B	0.9600
C9—C14	1.389 (2)	C28—H28A	0.9600
C9—C10	1.390 (2)	C28—H28C	0.9600
C10—C11	1.387 (2)	C29—H29C	0.9600
C10—H10	0.9300	C29—H29B	0.9600
C11—C12	1.383 (2)	C29—H29A	0.9600
C11—H11	0.9300	O7A—C25A	1.4652 (18)
C12—C13	1.389 (2)	C25A—H25A	0.9600
C13—C14	1.383 (2)	C25A—H25B	0.9600
C13—H13	0.9300	C25A—H25C	0.9600
C14—H14	0.9300	O7B—C25B	1.4642 (19)
C15—H15B	0.9600	C25B—H25D	0.9600
C15—H15C	0.9600	C25B—H25E	0.9600
C15—H15A	0.9600	C25B—H25F	0.9600
C16—C17	1.5243 (19)		
			
C3—O1—C28	117.37 (13)	C22—C17—C16	120.09 (13)
C1—O2—C29	117.97 (13)	C18—C17—C16	122.13 (13)
C5—O4—C7	107.90 (10)	C17—C18—C19	121.03 (16)
C12—O5—C15	117.50 (15)	C17—C18—H18	119.5
C26—O9—C27	116.34 (13)	C19—C18—H18	119.5
O2—C1—C6	122.80 (14)	C20—C19—C18	120.39 (16)
O2—C1—C2	114.41 (13)	C20—C19—H19	119.8
C6—C1—C2	122.78 (13)	C18—C19—H19	119.8
C3—C2—C1	120.13 (13)	C21—C20—C19	119.14 (16)
C3—C2—H2	119.9	C21—C20—H20	120.4
C1—C2—H2	119.9	C19—C20—H20	120.4
O1—C3—C2	125.39 (13)	C20—C21—C22	120.77 (16)
O1—C3—C4	116.06 (13)	C20—C21—H21	119.6
C2—C3—C4	118.54 (13)	C22—C21—H21	119.6
C5—C4—C3	118.67 (13)	C21—C22—C17	120.88 (15)
C5—C4—C8	107.82 (12)	C21—C22—H22	119.6
C3—C4—C8	133.49 (13)	C17—C22—H22	119.6
O4—C5—C6	121.85 (12)	C26—C23—C24	104.52 (11)
O4—C5—C4	113.69 (11)	C26—C23—C16	110.65 (12)
C6—C5—C4	124.46 (13)	C24—C23—C16	115.72 (12)
C5—C6—C1	115.31 (13)	C26—C23—H23	108.6
C5—C6—H6	122.3	C24—C23—H23	108.6
C1—C6—H6	122.3	C16—C23—H23	108.6
O4—C7—C9	109.10 (11)	O6B—C24—O6A	99.9 (4)
O4—C7—C16	107.85 (10)	O6B—C24—O7B	123.3 (4)
C9—C7—C16	115.55 (11)	O6A—C24—O7B	23.4 (3)
O4—C7—C8	104.70 (10)	O6B—C24—O7A	23.9 (4)
C9—C7—C8	106.01 (11)	O6A—C24—O7A	123.6 (2)
C16—C7—C8	113.05 (12)	O7B—C24—O7A	147.0 (3)
O3—C8—C4	131.22 (13)	O6B—C24—C23	131.9 (4)
O3—C8—C7	124.37 (12)	O6A—C24—C23	128.2 (2)
C4—C8—C7	104.38 (12)	O7B—C24—C23	104.8 (2)
C14—C9—C10	118.20 (13)	O7A—C24—C23	108.04 (14)
C14—C9—C7	122.03 (12)	O8—C26—O9	124.70 (14)
C10—C9—C7	119.65 (13)	O8—C26—C23	126.77 (13)
C11—C10—C9	121.33 (15)	O9—C26—C23	108.48 (12)
C11—C10—H10	119.3	O9—C27—H27B	109.5
C9—C10—H10	119.3	O9—C27—H27C	109.5
C12—C11—C10	119.91 (14)	H27B—C27—H27C	109.5
C12—C11—H11	120.0	O9—C27—H27A	109.5
C10—C11—H11	120.0	H27B—C27—H27A	109.5
O5—C12—C11	124.79 (15)	H27C—C27—H27A	109.5
O5—C12—C13	115.96 (15)	O1—C28—H28B	109.5
C11—C12—C13	119.25 (14)	O1—C28—H28A	109.5
C14—C13—C12	120.55 (15)	H28B—C28—H28A	109.5
C14—C13—H13	119.7	O1—C28—H28C	109.5
C12—C13—H13	119.7	H28B—C28—H28C	109.5
C13—C14—C9	120.73 (14)	H28A—C28—H28C	109.5
C13—C14—H14	119.6	O2—C29—H29C	109.5
C9—C14—H14	119.6	O2—C29—H29B	109.5
O5—C15—H15B	109.5	H29C—C29—H29B	109.5
O5—C15—H15C	109.5	O2—C29—H29A	109.5
H15B—C15—H15C	109.5	H29C—C29—H29A	109.5
O5—C15—H15A	109.5	H29B—C29—H29A	109.5
H15B—C15—H15A	109.5	C24—O7A—C25A	115.6 (2)
H15C—C15—H15A	109.5	C24—O7B—C25B	122.2 (4)
C17—C16—C23	111.16 (11)	O7B—C25B—H25D	109.5
C17—C16—C7	111.15 (11)	O7B—C25B—H25E	109.5
C23—C16—C7	111.99 (12)	H25D—C25B—H25E	109.5
C17—C16—H16	107.4	O7B—C25B—H25F	109.5
C23—C16—H16	107.4	H25D—C25B—H25F	109.5
C7—C16—H16	107.4	H25E—C25B—H25F	109.5
C22—C17—C18	117.78 (14)		
			
C29—O2—C1—C6	5.6 (2)	C11—C12—C13—C14	−1.4 (2)
C29—O2—C1—C2	−175.50 (14)	C12—C13—C14—C9	0.4 (2)
O2—C1—C2—C3	179.32 (14)	C10—C9—C14—C13	1.3 (2)
C6—C1—C2—C3	−1.7 (2)	C7—C9—C14—C13	177.15 (13)
C28—O1—C3—C2	−9.3 (2)	O4—C7—C16—C17	−69.31 (14)
C28—O1—C3—C4	170.16 (14)	C9—C7—C16—C17	168.34 (11)
C1—C2—C3—O1	178.22 (14)	C8—C7—C16—C17	45.95 (15)
C1—C2—C3—C4	−1.2 (2)	O4—C7—C16—C23	55.69 (14)
O1—C3—C4—C5	−176.09 (12)	C9—C7—C16—C23	−66.66 (15)
C2—C3—C4—C5	3.4 (2)	C8—C7—C16—C23	170.95 (11)
O1—C3—C4—C8	1.8 (2)	C23—C16—C17—C22	133.41 (13)
C2—C3—C4—C8	−178.72 (15)	C7—C16—C17—C22	−101.13 (14)
C7—O4—C5—C6	173.08 (12)	C23—C16—C17—C18	−47.31 (18)
C7—O4—C5—C4	−6.36 (15)	C7—C16—C17—C18	78.15 (17)
C3—C4—C5—O4	176.52 (12)	C22—C17—C18—C19	0.7 (2)
C8—C4—C5—O4	−1.89 (16)	C16—C17—C18—C19	−178.61 (15)
C3—C4—C5—C6	−2.9 (2)	C17—C18—C19—C20	−0.1 (3)
C8—C4—C5—C6	178.68 (13)	C18—C19—C20—C21	−0.3 (3)
O4—C5—C6—C1	−179.29 (12)	C19—C20—C21—C22	0.1 (3)
C4—C5—C6—C1	0.1 (2)	C20—C21—C22—C17	0.4 (2)
O2—C1—C6—C5	−178.88 (13)	C18—C17—C22—C21	−0.8 (2)
C2—C1—C6—C5	2.3 (2)	C16—C17—C22—C21	178.48 (13)
C5—O4—C7—C9	−101.91 (12)	C17—C16—C23—C26	−57.94 (15)
C5—O4—C7—C16	131.87 (11)	C7—C16—C23—C26	177.07 (11)
C5—O4—C7—C8	11.22 (13)	C17—C16—C23—C24	−176.55 (11)
C5—C4—C8—O3	−173.21 (14)	C7—C16—C23—C24	58.46 (15)
C3—C4—C8—O3	8.7 (3)	C26—C23—C24—O6B	121.0 (7)
C5—C4—C8—C7	8.64 (14)	C16—C23—C24—O6B	−117.0 (7)
C3—C4—C8—C7	−169.44 (15)	C26—C23—C24—O6A	−57.0 (4)
O4—C7—C8—O3	169.64 (13)	C16—C23—C24—O6A	65.0 (4)
C9—C7—C8—O3	−75.06 (16)	C26—C23—C24—O7B	−57.3 (4)
C16—C7—C8—O3	52.51 (18)	C16—C23—C24—O7B	64.7 (4)
O4—C7—C8—C4	−12.04 (13)	C26—C23—C24—O7A	119.1 (3)
C9—C7—C8—C4	103.26 (12)	C16—C23—C24—O7A	−119.0 (3)
C16—C7—C8—C4	−129.18 (12)	C27—O9—C26—O8	−8.3 (2)
O4—C7—C9—C14	5.46 (17)	C27—O9—C26—C23	169.40 (14)
C16—C7—C9—C14	127.14 (14)	C24—C23—C26—O8	110.02 (19)
C8—C7—C9—C14	−106.80 (15)	C16—C23—C26—O8	−15.2 (2)
O4—C7—C9—C10	−178.71 (11)	C24—C23—C26—O9	−67.62 (15)
C16—C7—C9—C10	−57.03 (17)	C16—C23—C26—O9	167.16 (12)
C8—C7—C9—C10	69.03 (15)	O6B—C24—O7A—C25A	6.4 (13)
C14—C9—C10—C11	−1.9 (2)	O6A—C24—O7A—C25A	−1.0 (6)
C7—C9—C10—C11	−177.88 (13)	O7B—C24—O7A—C25A	−3.8 (9)
C9—C10—C11—C12	0.9 (2)	C23—C24—O7A—C25A	−177.3 (3)
C15—O5—C12—C11	3.5 (2)	O6B—C24—O7B—C25B	−0.3 (11)
C15—O5—C12—C13	−176.57 (16)	O6A—C24—O7B—C25B	−1.2 (9)
C10—C11—C12—O5	−179.32 (15)	O7A—C24—O7B—C25B	4.6 (13)
C10—C11—C12—C13	0.8 (2)	C23—C24—O7B—C25B	178.2 (6)
O5—C12—C13—C14	178.68 (14)		
